# Transcriptome analysis of hepatopancreas of *Litopenaeus vannamei* after acute exposure to *Alexandrium pacificum*

**DOI:** 10.1371/journal.pone.0355978

**Published:** 2026-08-25

**Authors:** Fuyuan Zeng, Haiming Chen, Huijie Yang, Tong Li, Hui Huang, Shafira Citra Desrika Putri, Zhangxi Hu, Yulei Zhang

**Affiliations:** 1 College of Fisheries, Guangdong Ocean University, Zhanjiang, China; 2 Guangdong Provincial Key Laboratory of Aquatic Animal Disease Control and Healthy Culture, Zhanjiang, China; Tanta University Faculty of Agriculture, EGYPT

## Abstract

*Alexandrium pacificum* is a toxic species among red tide-forming organisms. Red tide outbreaks can cause seawater hypoxia and discoloration, which impair shrimp feeding and may facilitate toxin accumulation. Exposure to *A. pacificum* has been linked to oxidative stress and immune disruption in *Litopenaeus vannamei*, highlighting the need to understand the pathogenic mechanisms involved. To investigate the immune response induced in *L. vannamei* upon exposure to *A. pacificum*. Healthy shrimp (2.5 ± 0.5 g) were immersed in *A. pacificum* lysate (1.0 × 10^4^ cells·mL^-1^) or natural seawater (control). The hepatopancreas was collected for transcriptome sequencing 72 h post-exposure. Transcriptomic analysis identified 264 differentially expressed genes (DEGs) in the exposure group compared to the control, of which 185 were upregulated and 79 were downregulated. DEGs involved in signal transduction, oxidative phosphorylation, protease inhibitors, and ribosomal protein functions were well prominent. GO enrichment analysis showed that immune system processes, lysosomal-mediated cellular processes, and metabolic functions were significantly enriched. KEGG analysis identified enriched pathways including lysosome, C‑type lectin receptor signaling, peroxisome, drug metabolism, and neuroactive ligand‑receptor interactions. This study offers molecular insights into the molecular defense mechanisms of *L. vannamei* against *A. pacificum* toxicity.

## 1. Introduction

Red tide is a harmful ecological phenomenon in which specific environmental conditions induce high concentration or exponential proliferation of certain protozoa, phytoplankton, and bacteria in seawater, resulting in water discoloration [1]. Water discoloration reduces light penetration, which impairs feeding behavior in *L. vannamei* and disrupts primary production. Moreover, the discolored water serves as a visible indicator of potential toxin presence, signaling risks of toxin accumulation in shrimp and subsequent threats to human consumers via trophic transfer [2,3]. The continuous outbreak of red tides can endanger human health, causing headaches and problems in the gastrointestinal tract, throat, and even the respiratory system [4]. Red tide organisms are diverse, and most of them are toxic, with some being highly toxic. As one of the red tide species, Alexandrium releases paralytic shellfish toxins (PSTs) into seawater during its growth cycle, thereby posing potential threats to marine ecosystems and human health [5]. PSTs exert their toxicity by blocking voltage-gated sodium channels in nerve cells, leading to neuromuscular paralysis [6]. These toxins are water-soluble and heat-stable, readily accumulated in shellfish tissues and transferred along the food chain, with slow elimination rates posing prolonged risks to consumers [2,3]. Transcriptomic analysis of mussels exposed to *Alexandrium* revealed that PSTs upregulated endocytosis, lysosomal pathways, and immune-related signaling pathways [7]. Exposure of scallops to PSTs-producing dinoflagellates reduced gut microbiota diversity and disrupted community structure, impairing normal growth and survival [8].

*Litopenaeus vannamei*, also known as Pacific white shrimp, is one of the three most highly farmed shrimp species in the world. Its rapid growth rate, strong disease resistance, and ease of transportation make it an excellent variety for high-yield aquaculture [9,10]. Generally, under different environmental stresses, *L. vannamei* will experience varying degrees of negative impacts on its growth performance and immune capacity [11,12]. Exposure of *L. vannamei* to nitrite and microplastics caused gill deformities (including gill filament contraction, loosened gill filament connection, severe deformation of gill blood vessels) and vacuolization, adversely affecting gill physiological homeostasis [13]. Ammonia exposure can affect the antioxidant defense, endoplasmic reticulum stress, and apoptosis in Marsupenaeus japonicus, potentially disrupting the protein synthesis system under severe oxidative stress conditions [14]. Acute alkalinity stress can lead to damage in the gill structure of *L. vannamei*, affecting its ion transport function. Additionally, it results in the upregulation of genes related to ion transport proteins and induces apoptosis in gill cells [15]. Previous studies showed that *L. vannamei* encodes numerous stress-resistant factors at the molecular level to cope with various environmental and biological stresses [15]. Through RNA sequencing (RNA-seq), the overall transcriptional level of organisms under specific conditions can be studied, which helps to elucidate the molecular mechanisms of complex biological pathways and shape regulation networks [16].

In our previous study, acute exposure to A. pacificum caused multiple tissue lesions in L. vannamei, with the most common clinical signs being cell swelling and vacuolization in hepatopancreas, intestine, gill, muscle, and nerve tissues, accompanied by decreased antioxidant defense [17]. To investigate the toxicity of the toxic dinoflagellate *A. pacificum* to *L. vannamei* and the immune response of the shrimp. This study infected *L. vannamei* with *A. pacificum* through immersion, and performed transcriptome sequencing analysis on the hepatopancreas 72 h post-infection, differentially expressed genes were screened and subjected to GO and KEGG pathway enrichment analysis, to deeply explore the response mechanisms of differential genes and immune-related pathways in shrimp against toxic dinoflagellate infection. The present study was designed to (1) characterize the transcriptomic response of *L. vannamei* hepatopancreas following 72 h of acute exposure to A. pacificum, (2) identify key DEGs and enriched pathways associated with immune defense and detoxification, and (3) elucidate the molecular mechanisms underlying shrimp tolerance to PSTs. These findings provide a foundation for understanding crustacean detoxification strategies under red tide toxin stress. Although previous studies have investigated the toxic effects of *Alexandrium* species on bivalves, the molecular defense mechanisms in penaeid shrimp have received limited attention. This study provides a transcriptomic characterization of the hepatopancreas response in *L. vannamei* upon acute exposure to *A. pacificum*, with a focus on immune and detoxification pathways.

## 2. Materials and methods

### 2.1 Experimental materials

Healthy and active *L. vannamei* (body length: 5.3 ± 0.3 cm; weight: 2.5 ± 0.5 g) were obtained from the Donghai Island Biological Research and Experimental Base in Zhanjiang, Guangdong, China. Prior to the experiments, the shrimp were acclimated in the laboratory for one week to ensure a stable physiological condition. Only individuals showing uniform growth and high vitality were selected for the study. The experimental seawater was sourced from the same base, adjusted to a salinity of 25, and sterilized before use.

The dinoflagellate *A. pacificum* was purchased from Shanghai Guangyu Biotechnology Co., Ltd., China. The algae were cultured in f/2 medium using a programmable light incubator at 22°C with a salinity of 25. The light intensity was maintained at 30 μmol·m ⁻ ²·s ⁻ ¹ under a 14:10 h light/dark photoperiod. During the cultivation, the culture bottles were manually shaken three times daily at fixed intervals to ensure uniform nutrient distribution and growth.

All experiments were performed in the laboratory; no field collection of samples from distinct geographic areas was conducted; hence, a study area map is not provided.

### 2.2 Pacific alexandrium immersion exposure experiment

To determine the appropriate exposure concentration for the transcriptomic study, a preliminary dose-response analysis was conducted. The median lethal concentration (LC_50_) of *A. pacificum* for *L. vannamei* was estimated to be approximately 11,350 cells/mL over 72 h, calculated using the Karber method.

Based on this preliminary result, the officially exposed algal cell lysate was prepared according to the method of Yang et al. [18], with slight modifications: the *A. pacificum* culture was centrifuged at 4,000 r/min for 2 min at 4°C to separate the cells from the supernatant. The harvested algal cells were concentrated to 5.0 × 10^4^ cells/mL and then subjected to mechanical disruption using a tissue homogenizer at 70 Hz for 2 min with 0.5 mm diameter zirconium oxide beads. The resulting mixture was diluted five-fold with the previously collected supernatant to yield the final lysate for the subsequent trials.

For the exposure experiment, *L. vannamei* were partitioned into an experimental group and a control group, with three replicates per group, each replicate randomly stocked with 70 shrimps in 50 L of experimental water. The experimental group was exposed to the prepared *A. pacificum* lysate for 72 h, while the control group was maintained in sterilized natural seawater. Each group consisted of three biological replicates to ensure statistical reliability. No feeding occurred throughout the 72 h duration. At the end of the exposure period, six shrimp were randomly sampled from each replicate. The hepatopancreas tissues were excised from each shrimp individually and processed separately for RNA extraction, immediately flash-frozen in liquid nitrogen, and stored at −80°C for subsequent analysis.

### 2.3 RNA extraction, cDNA library construction, and sequencing

According to the method of Xu et al [19], RNA extraction, cDNA library construction and sequencing were evaluated and adjusted. Total RNA was extracted from the hepatopancreas of *L. vannamei* according to the instructions of the Trizol reagent kit, and genomic DNA was removed using DNase I to avoid contamination. The purity and quality of the RNA were initially assessed by 1% agarose gel electrophoresis. Subsequently, the integrity and concentration of the RNA were further tested using a Nano Photometer spectrophotometer and an Agilent 2100 Bioanalyzer to ensure that the RNA quality met the required standards. Qualified total RNA was then used for sequencing experiments. mRNA was purified using poly-T oligo-attached magnetic beads. The first cDNA strand was synthesized using random hexamer primers and M-MuLV reverse transcriptase (with RNase H). The second cDNA strand was synthesized with the catalysis of DNase I and RNase H. The synthesized cDNA fragments were purified using the AMPure XP system and then amplified by PCR to construct the cDNA library. Finally, the constructed cDNA library was sequenced on the Illumina platform, and the sequencing was performed by Biomarker Technologies Corporation in Beijing.

### 2.4 Transcriptome data analysis

After the raw data was obtained, adapter sequences and low-quality data were filtered to obtain clean data. HISAT2 was used to align the reads from the RNA sequencing experiment. The reads were assembled using StringTie, and the expression levels were normalized using FPKM (Fragments Per Kilobase of transcript per Million mapped reads) based on the maximum flow algorithm. DESeq2 was used to analyze the differential expression between samples based on the negative binomial distribution. DESeq2 was used to analyze the differential expression between samples using the negative binomial distribution. In detecting differentially expressed genes (DEGs), P-values < 0.05 and |Foldchange| > 1.5 were used as screening criteria. The average gene expression levels in the experimental group compared to the control group were calculated, and the differentially expressed genes were subjected to KEGG pathway enrichment analysis and GO enrichment analysis.

### 2.5 qPCR Validation of transcriptome

Seven differentially expressed genes were randomly selected for qPCR validation, including hormone receptor 4 (*hr4-like*), ras-related protein Rab-3 (*rpR3-like*), 2-acylglycerol O-acyltransferase 2-B (*2O2B-like*), transmembrane protein 198 (*tp198-like*), chitinase 1 precursor (*L9501*), pyruvate formate-lyase (*pf1-like*), and guanine deaminase (*gd-like*). qPCR validation was performed on these seven differentially expressed genes [20]. Primers were designed using Primer Premier 5.0 software (Table 1), and the accuracy of the RNA-seq data was verified by real-time quantitative PCR (qPCR). For the reverse transcription step, the HiScript II Q RT SuperMix for qPCR kit was used, and for the qPCR step, the ChainQ Universal SYBR qPCR Master Mix kit was employed. β-actin was used as the reference gene, and the relative expression levels of the target genes were calculated using the 2^-△△Ct^ method. To minimize experimental errors, each sample was subjected to three biological replicates and technical replicates, ensuring the precision and reproducibility of the experimental results.

**Table 1:** PCR primer pairs used in hepatopancreas.

Gene name	Forward Primer Sequence (5’-3’)	Reverse Primer Sequence (5’-3’)
*hr4-like*	TCAAGTGAGGGGAATGACGC	CCCTCCCGCTGCACTAATAC
*rpR3-like*	ATTCACCCAAGCCCTTTCGG	TGCAGACGATCAAGGCAGAG
*2O2B-like*	TCTTCAGATGGCACTGGATGC	TGGCGGAAACCCTTTCTTACT
*tp198-like*	GAAGTGTGAACCTCCAGCGA	AGGGAGGCAGAAGGCAGTAA
*L9501*	TCTTCCAGGTCGTTCACGC	ATCCTCCTTGTTCGGTGACG
*pf1-like*	GGTCGAGAGGTTTCCTGGAC	AATCTGGGGCAATGGTCTGT
*gd-like*	ACCCAAATGCACACAAAACCAA	GGAGTAGATGAGGTCGGTGC
*β-actin*	CCACGAGACCACCTACAAC	AGCGAGGGCAGTGATTTC

## 3. Results

### 3.1 Sequencing data quality and overview of DEGs

**Fig 1:**
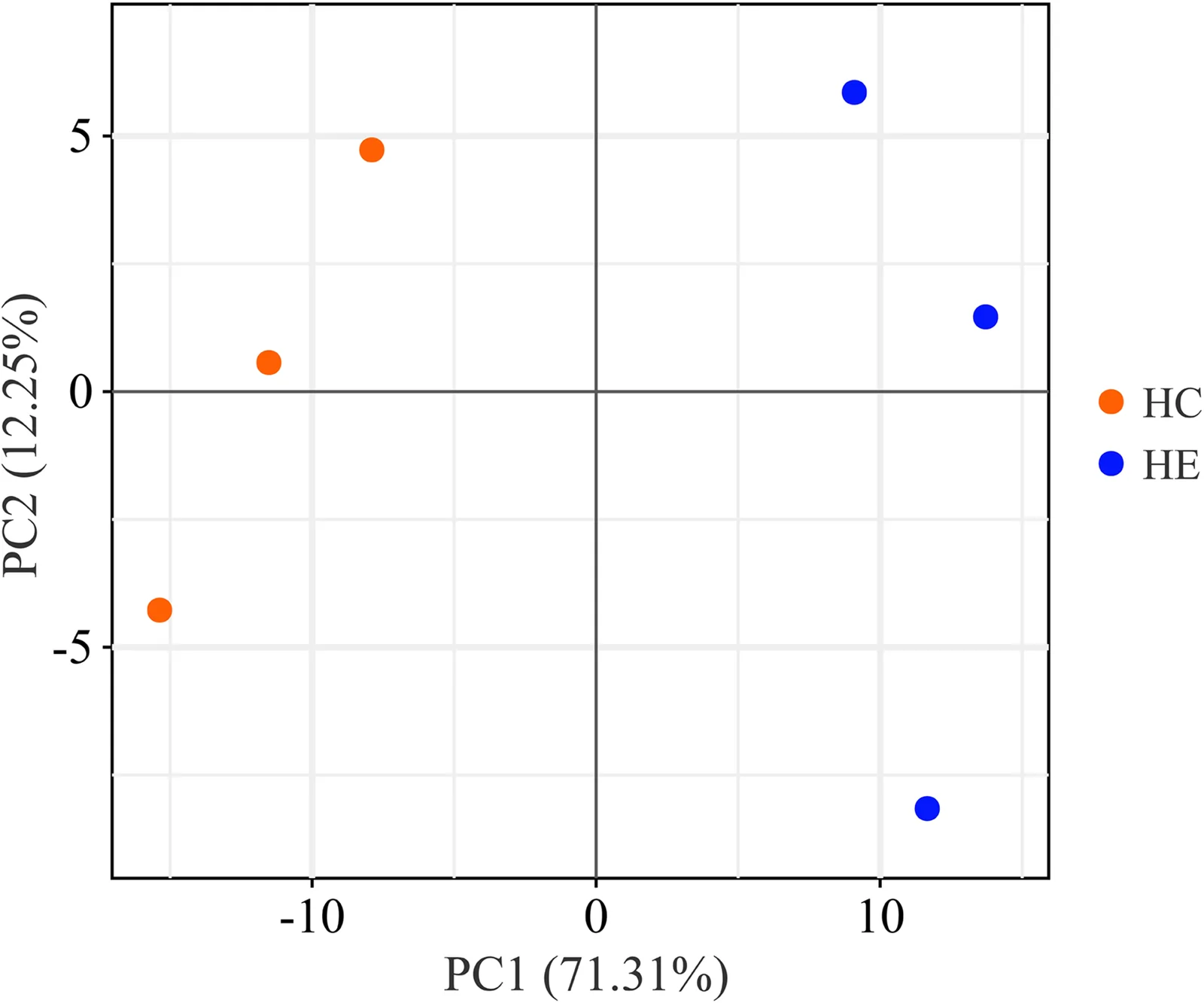
PCA plot of gene expression profiles in the hepatopancreas. Each point represents a biological replicate (n = 3 per group).

High-throughput sequencing of the hepatopancreas tissues of *L. vannamei* was performed on the Illumina HiSeq 2000 platform, generating over 10 Gb of raw data for per sample. After quality filtering, 241,809,875 clean reads were obtained. As shown in Table 2, the GC content ranged from 56.09% to 62.70%, Q20 ≥ 97.92%, and Q30 ≥ 94.05% across all samples. Principal component analysis (PCA) based on gene expression profiles (Fig 1) showed that the three biological replicates within each group clustered closely together, while the control and exposure groups were clearly separated along PC1 (71.31% of total variance), confirming the reproducibility and reliability of the sequencing data.

**Table 2:** Summary of RNA‑seq data quality after filtration.

Samples		Clean reads No.	Clean bases (bp)	GC(%)	Q20(%)	Q30(%)
Control Group	HC1	34,295,763	10,254,758,114	59.07	97.94	94.05
	HC2	41,805,726	12,489,154,418	58.36	98.46	95.46
	HC3	44,191,865	13,180,675,040	56.09	98.52	95.62
Algal Exposure Group	HE1	47,604,649	14,089,530,184	62.70	97.92	94.20
	HE2	39,550,139	11,822,653,242	61.13	98.06	94.33
	HE3	34,361,733	10,249,703,350	62.96	98.07	94.36

**Fig 2:**
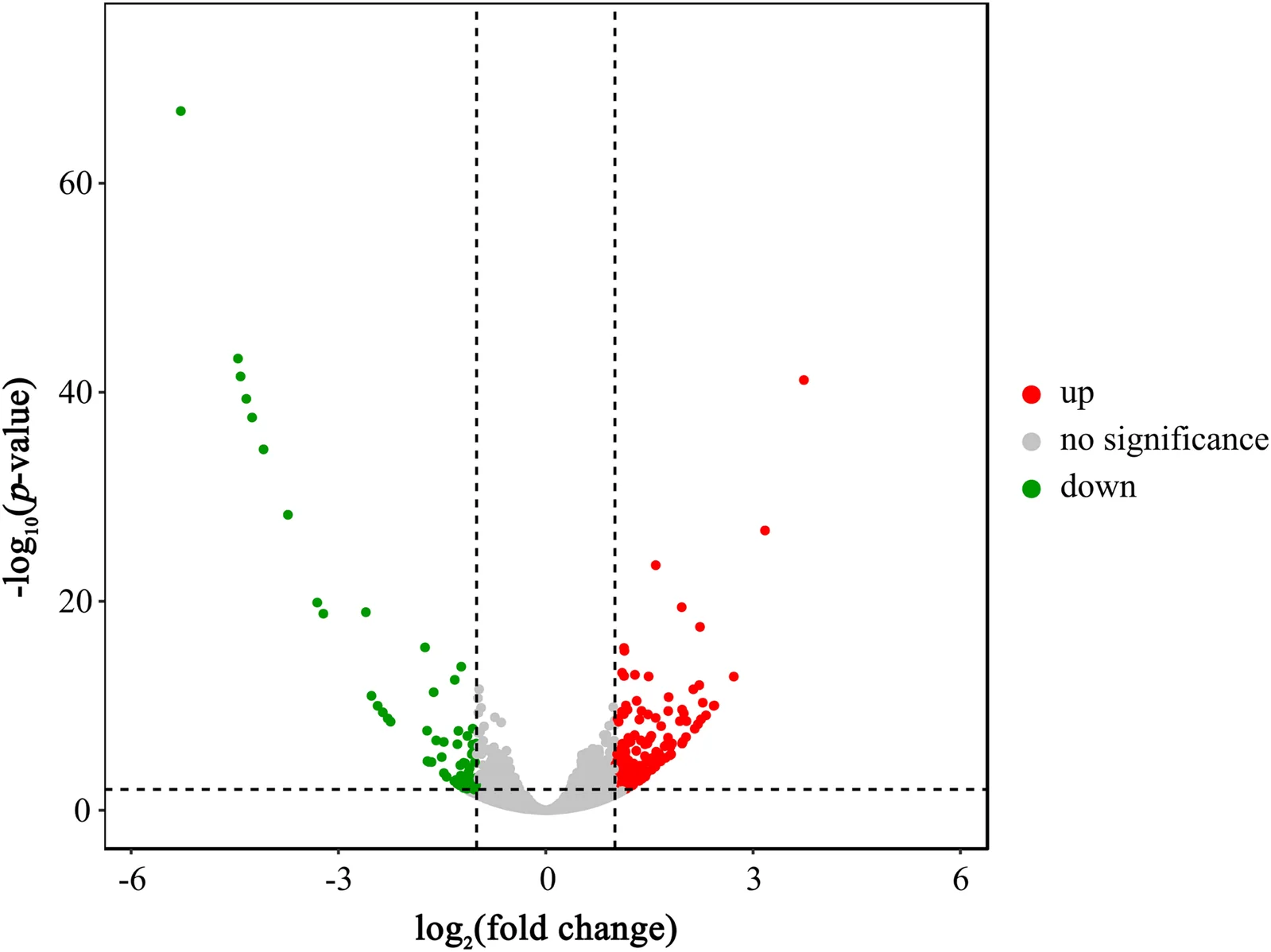
Volcano plot of DEGs in hepatopancreas. Red and green dots represent upregulated and downregulated genes, respectively; gray dots indicate non-significant genes.

Using the criteria of *p* < 0.05 and |Fold change| > 1.5, a total of 264 DEGs were identified, of which 185 were upregulated and 79 downregulated (Fig 2).

### 3.2 GO enrichment analysis

**Fig 3:**
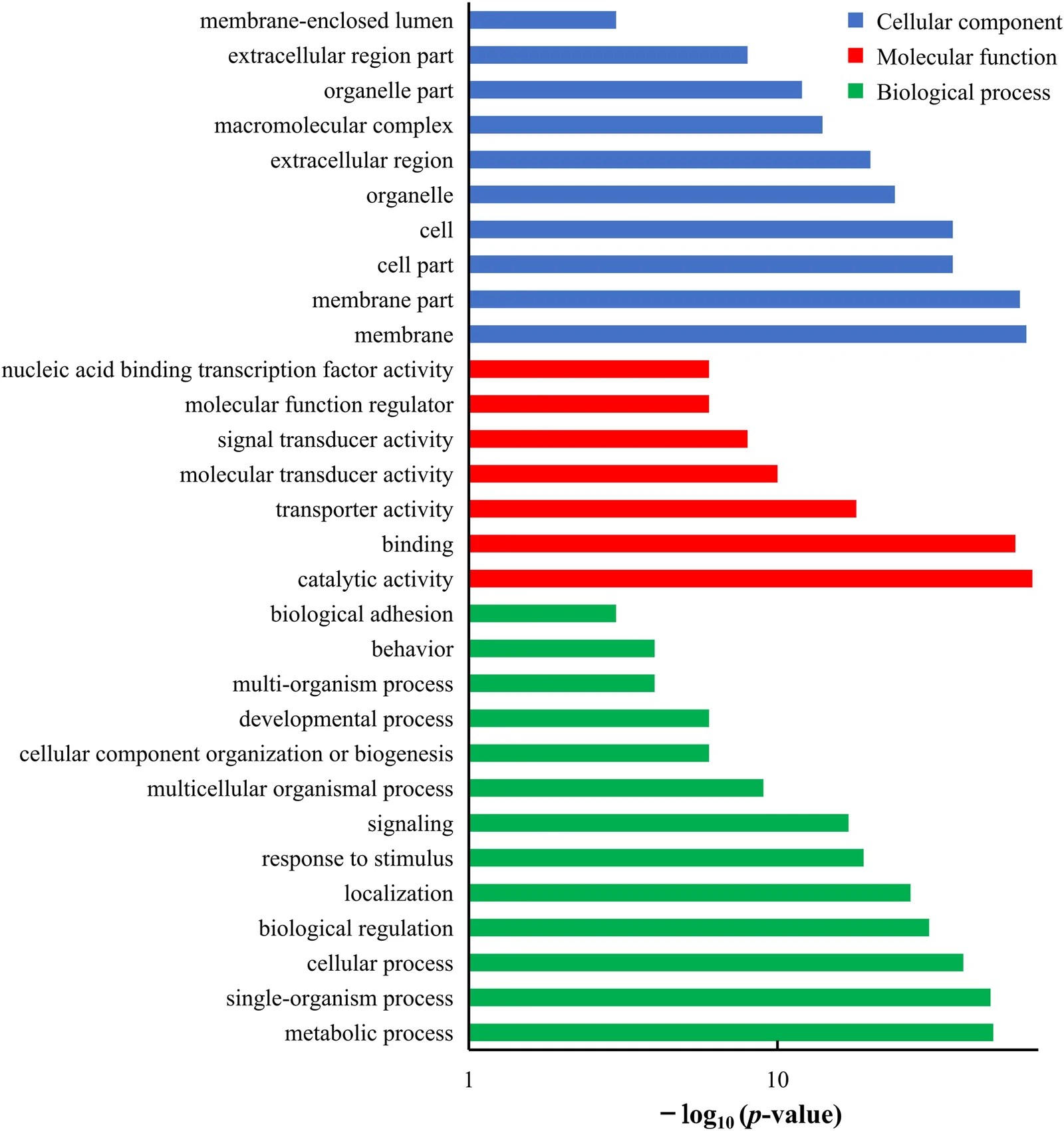
GO enrichment analysis of DEGs in the hepatopancreas.

To further analyze the functions of differentially expressed genes in the hepatopancreas of *L. vannamei* after exposure to *A. pacificum*, GO classification and enrichment analysis were performed on the DEGs from both the experimental and control groups (Fig 3; top 20 GO terms with the smallest *p*-values are shown). Among the three main categories, the most significantly enriched terms were: membrane and membrane part (Cellular Component), catalytic activity and binding (Molecular Function), and metabolic processes, single‑organism processes, and cellular processes (Biological Process).

### 3.3 KEGG enrichment analysis

**Fig 4:**
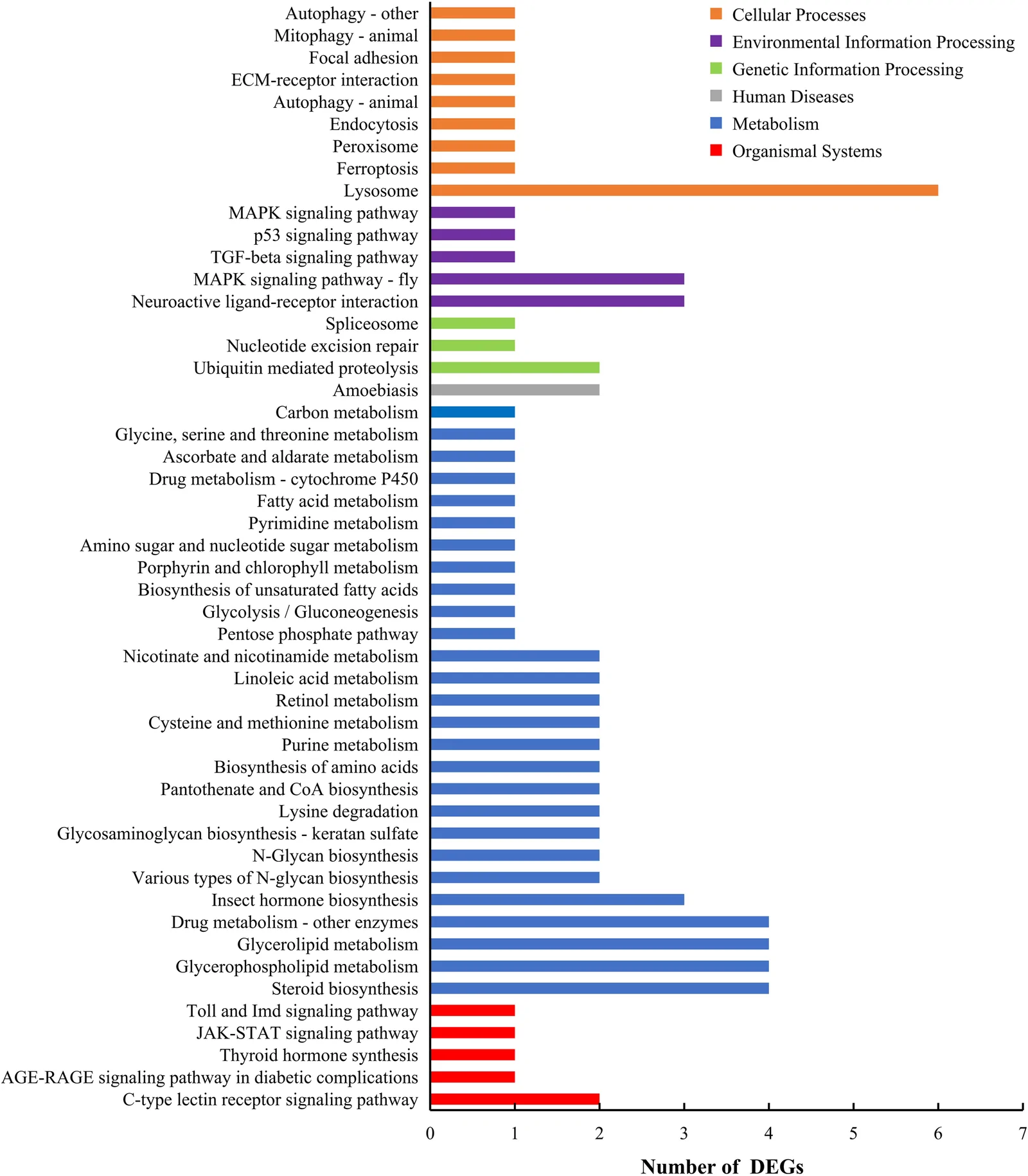
KEGG pathway enrichment analysis of DEGs in hepatopancreas.

KEGG enrichment analysis (Fig 4) showed that DEGs were enriched across multiple functional categories. Within cellular processes, the most significantly enriched pathway was lysosome. For Metabolism, DEGs were enriched in drug metabolism, glycerolipid metabolism, glycerophospholipid metabolism, steroid biosynthesis, and insect hormone biosynthesis. In the category of Environmental Information Processing, the MAPK signaling pathway and neuroactive ligand‑receptor interaction were the predominant enriched pathways.

### 3.4 Immune- and metabolism-related DEGs

From the significantly enriched KEGG pathways, 17 DEGs potentially involved in the shrimp’s response to *A. pacificum* toxins were selected (Table 3). These genes fell into four functional categories. Of these, three genes involved in Recognition and Early Response—C‑type lectin domain family 17 member A, phenoloxidase‑activating factor‑1, and hemolymph clottable protein—were all upregulated. The Modulation and Protection category included six genes: dipeptidyl peptidase, legumain, heat shock 70 kDa protein 12A, ETS homologous factor, and ankyrin repeat domain‑containing protein 1 isoform X1 were upregulated, whereas lysosome membrane protein 2 and ras‑related protein Rab‑3 were downregulated. For Metabolic Reprogramming, five genes were identified: gamma‑butyrobetaine dioxygenase, low‑density lipoprotein receptor, and gastric triacylglycerol lipase were upregulated, while argininosuccinate synthase and pancreatic lipase‑related protein 2 were downregulated. The remaining two genes, grouped as Energy and Structure, comprised cytoplasmic dynein 2 light intermediate chain (upregulated) and transmembrane protein 198 (downregulated).

**Table 3:** DEGs involved in immune and metabolic responses in the hepatopancreas.

Gene ID	Function	*P*-value	log2(fold changes)	Fold change	Regulated
I. Recognition and Early Response					
gene-27511	C-type lectin domain family 17, member A	1.07×10^-13^	1.2902	2.45	up
gene-23275	phenoloxidase-activating factor 1	1.60×10^-13^	2.7200	6.59	up
gene-19745	hemolymph clottable protein	8.52×10^-6^	1.1548	2.23	up
II. Modulation and Protection					
gene-27616	dipeptidyl peptidase	3.30×10^-3^	1.0029	2.00	up
gene-27864	legumain	1.26×10^-7^	1.5109	2.85	up
gene-03317	heat shock 70 kDa protein 12A	1.70×10^-3^	1.2433	2.37	up
gene-20924	ETS homologous factor	2.00×10^-4^	1.4194	2.67	up
gene-28835	ankyrin repeat domain-containing protein 1 isoform X1	4.80×10^-3^	1.0014	2.00	up
gene-06181	lysosome membrane protein 2	6.00×10^-4^	−1.1939	2.29	down
gene-22611	ras-related protein Rab-3	5.50×10^-3^	−1.0211	2.03	down
III. Metabolic Reprogramming					
gene-15268	argininosuccinate synthase	2.40×10^-8^	−1.7177	3.29	down
gene-28035	gamma-butyrobetaine dioxygenase	1.67×10^-27^	3.1723	9.01	up
gene-10940	low-density lipoprotein receptor	7.45×10^-8^	1.5259	2.87	up
gene-01959	gastric triacylglycerol lipase	9.26×10^-11^	2.4385	5.42	up
gene-10077	pancreatic lipase-related protein 2	3.29×10^-9^	−2.2457	4.47	down
IV. Energy and Structure					
gene-10078	transmembrane protein 198	1.70×10^-3^	−1.0175	2.02	down
gene-04407	cytoplasmic dynein 2 light intermediate chain	1.37×10^-5^	1.4576	2.75	up

**Fig 5:**
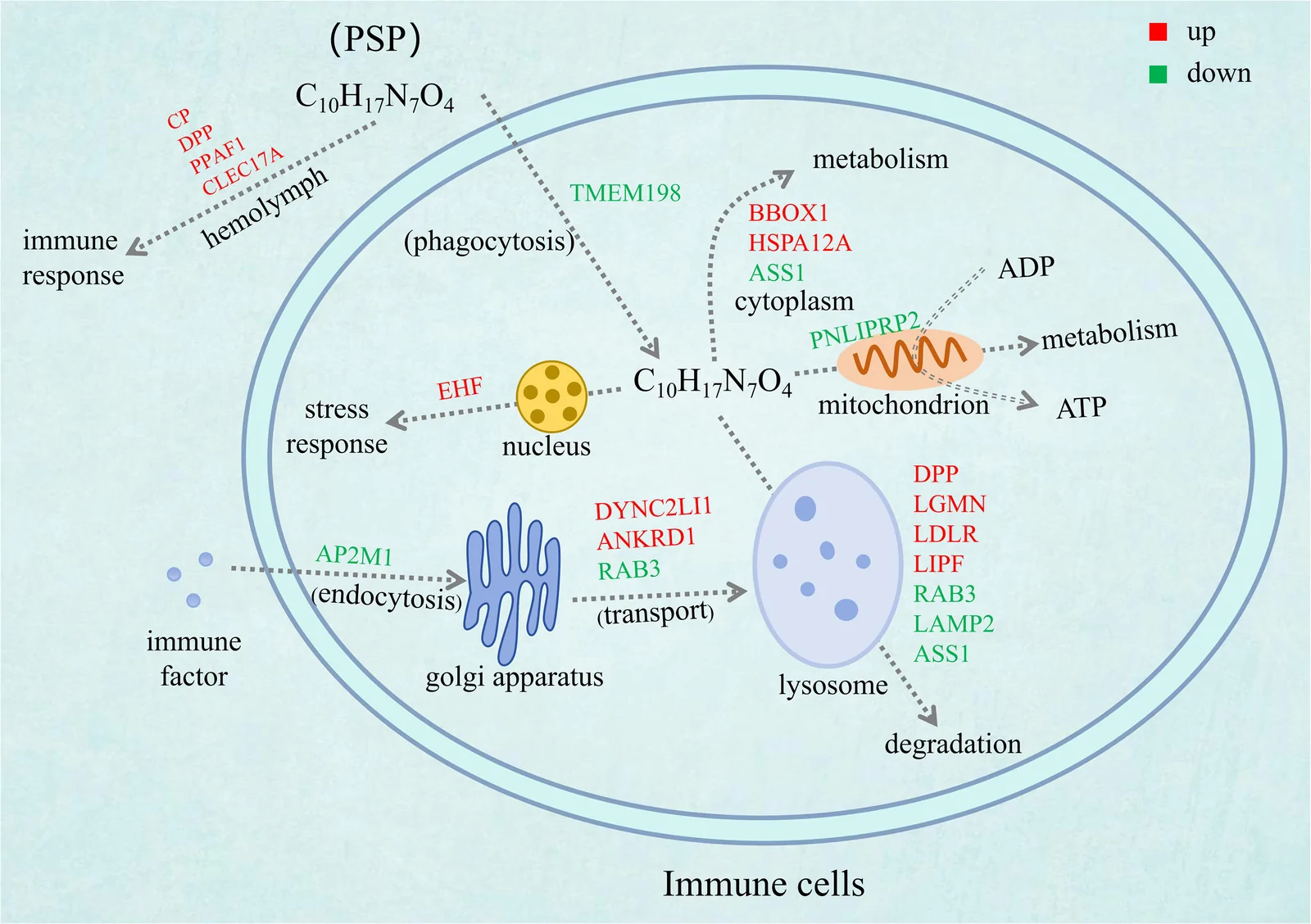
Schematic diagram of PSP metabolism in immune cells. Red and green indicate up and downregulated genes, respectively.

The cellular localization and regulatory direction of these selected genes are presented in Fig 5. Genes participating in endocytosis, lysosomal processing, and mitochondrial metabolism were mapped onto the PSP detoxification network; upregulated genes are marked in red and downregulated genes in green.

### 3.5 qPCR validation

**Fig 6:**
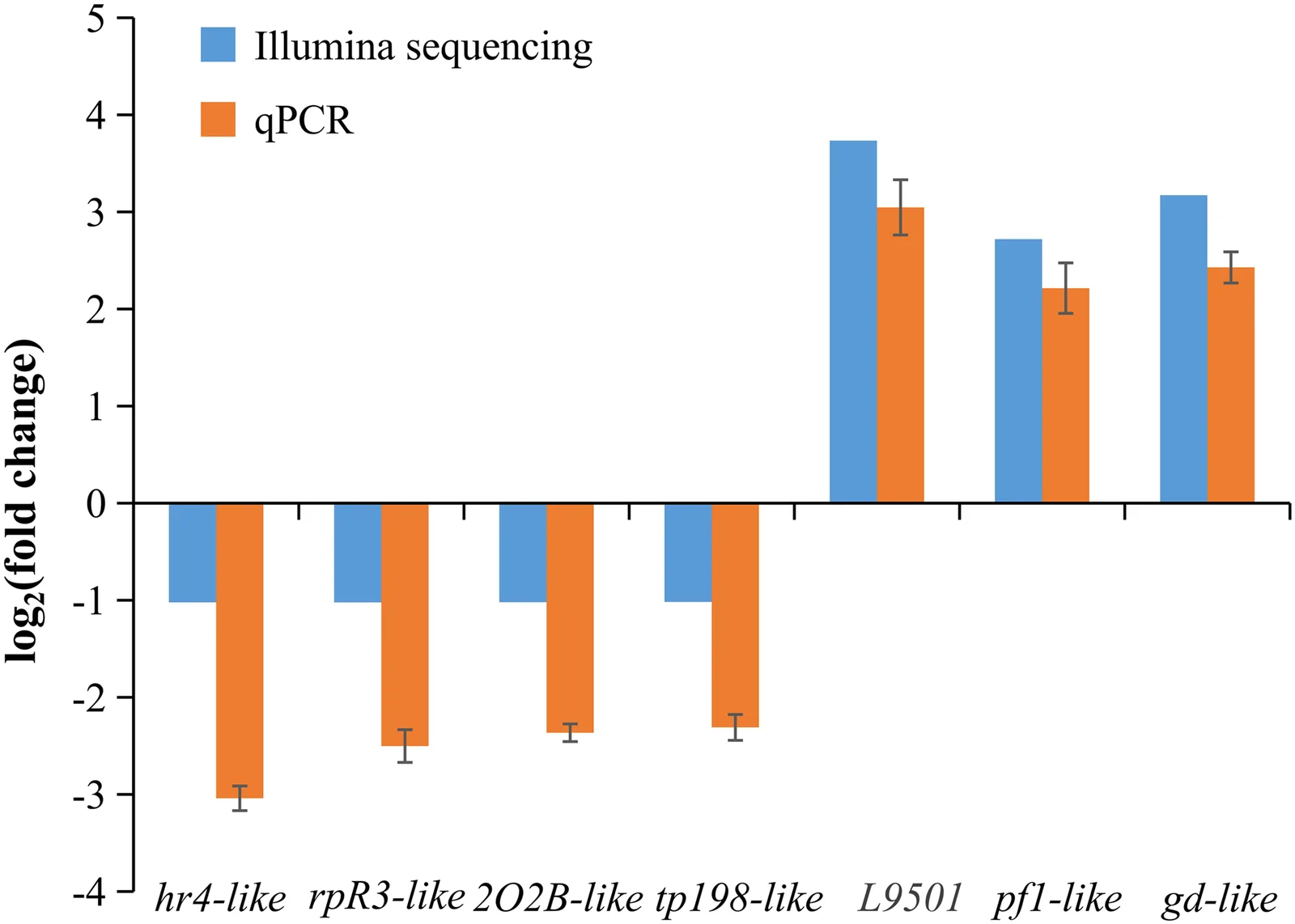
Comparison of the expressions of DEGs determined by Illumina sequencing and qPCR.

To validate the transcriptome sequencing results, seven DEGs were selected for qPCR analysis, including three upregulated genes (*L9501*, *pfl-like*, *gd-like*) and four downregulated genes (*hr4-like*, *rpR3-like*, *2O2B-like*, *tp198-like*). The log_2_(fold change) values obtained from RNA‑seq and qPCR were as follows: *hr4-like* (−1.02 vs −3.04), *rpR3-like* (−1.02 vs −2.05), *tp198-like* (−1.02 vs −2.31), *pf1-like* (2.72 vs 2.22), *L9501* (3.73 vs 3.05), *gd-like* (3.17 vs 2.43), and *2O2B-like* (−1.02 vs −2.37). Despite numerical discrepancies attributable to technical differences between the two platforms, the expression trends of all seven genes were consistent across methods (Fig 6), confirming the reliability of the RNA‑seq data.

## 4. Discussion

The paralytic shellfish toxins produced by *A. pacificum* cause significant losses worldwide each year and are among the most harmful marine biotoxins to humans [21]. Under specific environmental conditions, such as a salinity of 25, using 1 L of culture medium, and reducing nutrient concentrations to one-fourth of the standard amount, *Alexandrium tamarense* can achieve maximum toxin production and release, posing a greater threat to marine life and human health [22]. When shrimp are exposed to these toxins, their oxidative-antioxidative balance system may be disrupted, leading to free radicals accumulation. This can cause lipid peroxidation in gill tissues and activate cellular stress responses, including upregulated of Caspase gene expression, which may be associated with increased programmed cell death. This findings indicated the potential threat of *A. tamarense* toxins to shrimp health and survival [23]. Investigating the toxic effects of *A. pacificum* on *L. vannamei* is crucial for understanding the molecular mechanisms of marine organisms’ responses to such toxins and for developing strategies to mitigate the impacts of harmful algal blooms on aquaculture and marine ecosystems.

### 4.1 Pattern-recognition and immediate effector genes

Upon invasion by dinoflagellate toxins, *L. vannamei* rapidly activates a series of immune cascade reactions. These include hemocyte phagocytosis, encapsulation, aggregation, and the induction of the humoral immune system, which includes hemolymph defense functions, immune factors like lectins, and regulatory factors such as the prophenoloxidase activation system [24]. This immune response is crucial for shrimp to counteract the harmful effects of dinoflagellate toxins, such as those produced by *A. pacificum*, which can cause physiological changes and even death in aquatic animals [18]. As illustrated in the immune pathway (Fig 5), the hemolymph immune response to dinoflagellate toxins involved key components: C-type lectin, phenoloxidase-activating factor 1, and hemolymph clottable protein, all of which were up-regulated.

C-type lectins recognize pathogens, promote their aggregation, and enhance immune cell clearance, helping hosts combat infections while ensuring the immune specificity and effectiveness [25]. Previous studies have shown that C-type lectins in *L. vannamei* and Fenneropenaeus chinensis are significantly upregulated following infection with Vibrio anguillarum, Staphylococcus aureus, and Vibrio parahaemolyticus [26,27]. Similarly, in swimming crabs, C-type lectin levels in hemocytes rise significantly after *V. alginolyticus* challenge [28]. In this study, the significant up-regulation of C-type lectin suggests that it may enhance the immune defense of shrimp against *Alexandrium* toxins by recognizing and binding to glycosylated receptors on the toxin surface, promoting aggregation and subsequent clearance [29]. Hemolymph clottable protein, a key component of the crustacean immune system that prevents blood loss during injury and defends against pathogen invasion [30], was also significantly up-regulated. This suggests that it may enhance the shrimp immune response to Alexandrium toxins by forming clots to prevent toxin spread and by providing a stable environment for immune cells, thereby improving the efficiency of the immune reaction. Similarly, phenoloxidase-activating factor 1 (*POAF-1*), which activates prophenoloxidase (*proPO*) to convert into active phenoloxidase (*PO*) and thereby participates in host immune defense [31], was upregulated in this experiment, indicating its regulatory role in the immune response. Taken together, the up-regulation of C-type lectin, hemolymph clottable protein and *POAF-1* sets up a rapid, coordinated first line of defence: lectins label the toxins, the forming clot slows their spread, and *POAF-1* initiates the serine-protease cascade that arms downstream effectors—thereby turning early recognition into a prompt, locally focused containment.

### 4.2 Immune modulation and cellular protection related genes

To prevent an uncontrolled inflammatory burst, shrimp fine-tune the response through a second layer of regulators. Dipeptidyl peptidase IV (*DPP IV/CD26*), markedly up-regulated after *A. pacificum* exposure, is a serine protease that cleaves pro-inflammatory chemokines and simultaneously triggers apoptosis of irreversibly damaged hemocytes [32]. Its dual role—signal quenching and cell clearance—helps confine the toxin-induced lesion without collateral tissue damage. Legumain, also elevated, further accelerates lysosomal proteolysis of endocytosed toxins, echoing the well-documented lysosome-mediated detoxification in crustaceans [33]. Heat shock 70 kDa protein 12A (*Hsp70-12A*) acts as a molecular chaperone that refolds stress-denatured proteins and stabilises signalling complexes [34,35]. Ankyrin repeat domain-containing protein 1 (*ANKRD1*), additionally induced, is up-regulated upon viral or bacterial infection and functions as a feedback repressor of NF-κB/MAPK signalling [36,37]. Its comparable up-regulation following Alexandrium toxin exposure in the present study indicates that shrimp recruit this checkpoint to fine-tune the ensuing transcriptional programme, probably scaffolding NF-κB and MAPK signalling to sharpen the specificity of the induced gene set.

Conversely, lysosome membrane protein 2 (*LIMP-2*) and ras-related protein Rab-3-like are both down-regulated. Here, *Alexandrium* toxins trigger its down-regulation, implying a re-routed flux toward toxin degradation. Likewise, *Rab-3-like* is suppressed in *L. vannamei* within 6–24 h of white spot syndrome virus infection, prioritising immune granule exocytosis [38]. The corresponding reduction observed in this study supports the hypothesis that toxin stress redirects intracellular trafficking towards immediate immune granule release in crustaceans.

### 4.3 Metabolic reprogramming genes

To sustain the energetic and biosynthetic demands of the immune burst triggered by Alexandrium toxins, *L. vannamei* orchestrates a comprehensive metabolic reprogramming that prioritizes immediate defense over growth. A central feature of this adaptation is the strategic redirection of nitrogen metabolism, specifically regarding arginine allocation. Argininosuccinate synthase (*ASNS*) was markedly down-regulated in *Alexandrium*-exposed shrimp. This suppression parallels observations in *M. japonicus* challenged with Vibrio spp [39] and *L. vannamei* under environmental stress [40], where decreased *ASNS* activity diverts arginine away from the urea cycle and toward the inducible nitric oxide synthase (*iNOS*) pathway, facilitating the production of nitric oxide (*NO*), thereby enhancing NO-dependent antitoxic and bactericidal activities during acute toxin exposure [41].

This nitrogen re-routing is coupled with a significant reconfiguration of lipid metabolism to fuel cellular defenses. The up-regulation of gamma-butyrobetaine dioxygenase (*GBBD*) likely increases carnitine availability, facilitating mitochondrial β-oxidation and the rapid ATP generation necessary for phagocyte activity and ROS production [42]. Concurrently, the elevated expression of the low-density lipoprotein receptor (*LDLR*) suggests enhanced uptake of exogenous cholesterol and fatty acids, providing essential precursors for hemocyte membrane remodeling and lipid ligands for immune signaling [43]. Such functional prioritization is further evidenced by the reciprocal regulation of digestive lipases: the significant down-regulation of pancreatic lipase-related protein 2 (*LRP2*) suggests a temporary sacrifice of dietary lipid digestion to conserve energy [44], while the marked up-regulation of gastric triacylglycerol lipase (*TGL*) serves to mobilize stored triacylglycerols as immediate fuel [45]. These lipid metabolites also function as signaling molecules that link the metabolic state to immune gene expression via transcription factors such as PPARs [37].

These specialized shifts are ultimately supported by the intensification of energy production pathways. KEGG enrichment analysis identified significant activation of oxidative phosphorylation, drug metabolism, and glycerophospholipid metabolism. Notably, a high proportion of the most significantly differentially expressed genes were dedicated to oxidative phosphorylation, the primary mechanism for cellular ATP production [46]. This coordinated up-regulation ensures that the high energetic costs of phagocytosis, detoxification, and material transport are met through the efficient utilization of metabolic substrates [47,48]. Furthermore, the enrichment of glycerophospholipid metabolism suggests a critical role in maintaining membrane integrity and supporting signal transduction during the immune response [49]. Additionally, the involvement of drug metabolism pathways, particularly enzymes like carboxylesterases, underscores the active detoxification of toxin-derived compounds to mitigate physiological damage [50]. Collectively, the differential expression of ASNS, GBBD, LDLR, and LRP2 constitutes a sophisticated metabolic reprogramming strategy. By channeling resources toward immediate defense and membrane stability, *L. vannamei* maintains cellular homeostasis and enhances its survival capacity against the physiological challenges imposed by dinoflagellate toxins.

## 5. Conclusions

Transcriptomic analysis of *L. vannamei* hepatopancreas following *A. pacificum* exposure revealed 264 differentially expressed genes, with significant enrichment in lysosomal degradation, C-type lectin receptor signaling, and drug metabolism pathways. The shrimp mounted a coordinated defense involving immune recognition and lysosomal degradation. Energy metabolism was also adjusted, with increased lipid mobilization and nitric oxide production supporting the physiological demands of detoxification. Several immune- and metabolism-related genes identified here may serve as candidate markers for assessing toxin stress in shrimp culture. Their functional roles in conferring resistance to paralytic shellfish toxins warrant further investigation.

## Supporting information

S1 Table A comprehensive list of all abbreviations and their corresponding full forms appearing in the main text. (DOCX)
