# Supplementary material for: Transcriptome analysis of hepatopancreas of Litopenaeus vannamei after acute exposure to Alexandrium pacificum
Source: PLoS One. 2026 Aug 25;21(8):e0355978. doi: 10.1371/journal.pone.0355978 (PMC13505907; doi:10.1371/journal.pone.0355978)
Supplement: Supplementary file 1 [file pone.0355978.s001.docx]

Table S1. List of Abbreviations

| Abbreviations | Full names |
| --- | --- |
| 2O2B-like | 2-acylglycerol O-acyltransferase 2-B |
| ANKRD1 | ankyrin repeat domain-containing protein 1 isoform X1 |
| ASNS | argininosuccinate synthase |
| C-type lectin | C-type lectin domain family 17, member A |
| DEGs | differentially expressed genes |
| DPPIV/CD26 | dipeptidyl peptidase |
| GBBD | gamma-butyrobetaine dioxygenase |
| gd-like | guanine deaminase |
| GO | Gene Ontology |
| hr4-like | hormone receptor 4 |
| Hsp70-12A | heat shock 70 kDa protein 12A |
| iNOS | nitric oxide synthase |
| KEGG | Kyoto Encyclopedia of Genes and Genomes |
| L9501 | chitinase 1 precursor |
| LAMP-2 | lysosome membrane protein 2 |
| LDLR | low-density lipoprotein receptor |
| LRP2 | pancreatic lipase-related protein 2 |
| NO | nitric oxide |
| pf1-like | pyruvate formate-lyase |
| PO | phenoloxidase |
| POAF-1 | phenoloxidase-activating factor 1 |
| proPO | prophenoloxidase |
| PST | paralytic shellfish toxins |
| qPCR | quantitative PCR |
| rpR3-like | ras-related protein Rab-3 |
| TGL | gastric triacylglycerol lipase |
| tp198-like | transmembrane protein 198 |
